# Supplementary material for: Dental wear and oral pathology among sex determined Early Bronze-Age children from Franzhausen I, Lower Austria
Source: PLoS One. 2023 Feb 7;18(2):e0280769. doi: 10.1371/journal.pone.0280769 (PMC9904482; doi:10.1371/journal.pone.0280769)
Supplement: Attachment — Submitted filename: Response to Reviewers.docx [file pone.0280769.s003.docx]

# Response to reviewers

We thank the editor and reviewer for their encouraging feedback and suggested minor corrections. All corrections have been made following your recommendations, most notably we have removed the paragraph on weaning in the discussion as suggested by reviewer 1. Two references have also now been correctly inserted into the reference list and numbered (see reference 63 and 64). We thank you once again for helping us improve our manuscript.
